# Bispectral Index Monitoring Effect on Delirium Occurrence and Nursing Quality Improvement in Post-anesthesia Care Unit Patients Recovering From General Anesthesia: A Randomized Controlled Trial

**DOI:** 10.7759/cureus.66348

**Published:** 2024-08-07

**Authors:** Yi'an Huang, Lihua Huang, Jianhong Xu, Yangjuan Bao, Ying Qu, Yanzi Huang

**Affiliations:** 1 Department of Nursing, First Affiliated Hospital, Zhejiang University School of Medicine, Yiwu, CHN; 2 Department of Nursing, Fourth Affiliated Hospital of School of Medicine, International School of Medicine, International Institutes of Medicine, Zhejiang University, Huangzhou, CHN; 3 Department of Nursing, First Affiliated Hospital, Zhejiang University School of Medicine, Hangzhou, CHN; 4 Department of Anesthesiology, Fourth Affiliated Hospital of School of Medicine, International School of Medicine, International Institutes of Medicine, Zhejiang University, Yiwu, CHN; 5 Department of Nursing, Fourth Affiliated Hospital of School of Medicine, International School of Medicine, International Institutes of Medicine, Zhejiang University, Yiwu, CHN

**Keywords:** nursing quality, postoperative delirium, bispectral index, depth of anesthesia, post-anesthesia care unit

## Abstract

Background: The effect of intraoperative anesthesia depth monitoring on delirium occurrence and improvement of nursing quality in the post-anesthesia care unit (PACU) remains unclear. We aimed to explore the effect of intraoperative anesthesia bispectral index (BIS) monitoring on delirium occurrence and improvement of nursing quality in the PACU for patients recovering from general anesthesia.

Methods: This randomized controlled trial included 120 patients, aged 20-80 years, classified as grades I-III according to the American Society of Anesthesiologists. The BIS-guided group (group B) underwent intraoperative monitoring of BIS anesthesia depth (maintained within the anesthetic range (40-60)). The depth of anesthesia was not monitored in the non-BIS-guided group (group C). The patient’s vital signs were recorded at the beginning of the operation (T0), upon entering the PACU (T1), 15 min after extubation (T2), and after leaving the PACU (T3). Delirium score, emergence period (extubation and PACU observation times), and adverse events in the PACU were monitored. The nursing activity score (NAS) was used to evaluate the quality of care.

Results: Group B exhibited significantly lower heart rate and mean arterial pressure at T1 and T2, shorter time to extubation and PACU observation time, and a significantly lower incidence of adverse events than group C. Group B had significantly lower Ricker sedation-agitation scores and a lower incidence of delirium than group C. The NAS was significantly lower for group B than for group C. Patients aged 60−80 years in group C experienced agitation, requiring 30% more frequent assistance from one or two nurses than those in group B.

Conclusion: Intraoperative BIS monitoring can reduce the incidence of adverse events in the PACU, diminish the incidence of delirium during the recovery period in elderly patients, lessen the nursing workload, improve nursing quality, and promote patient rehabilitation, thus meriting clinical application.

## Introduction

Enhanced recovery after surgery (ERAS) currently stands as the focal point in surgical anesthesia research [1,2]. It represents an overall perioperative approach to reduce recovery time, although specific patient care is at the discretion of the physician. Therefore, ERAS protocols are mostly used during ambulatory procedures to ensure expedient discharge [3]. The post-anesthesia care unit (PACU) is used to closely monitor and treat patients' post-anesthesia until their vital signs stabilize, forming the basis of ERAS practice [4]. Because of pain post-surgery, residual effects of anesthetic drugs, and unrecovered protective reflexes, patients recovering from general anesthesia are prone to various life-threatening complications [5,6]. However, the large workload, heightened risks, fast-paced work environment, intensive technical operations, and dynamic nature of patient conditions keep PACU staff in a state of heightened physical and mental tension. Therefore, it is crucial to improve the quality management of PACU medical care and ensure the postoperative safety and comfort of patients [7,8].

Postoperative delirium is a complication affecting patients after surgery. Delirium occurring during the recovery period from general anesthesia prolongs the mechanical ventilation and intensive care unit (ICU) stay time of patients and increases the risk of postoperative complications and mortality [9-11]. One study has shown that 30-40% of postoperative delirium cases can be prevented [12]. Intraoperative anesthesia depth monitoring can reflect a patient’s anesthesia and sedation depth, guide the dosage of individual anesthesia drugs to decrease the recovery period and reduce the occurrence of postoperative complications [13]. However, the effect of intraoperative anesthesia depth monitoring on the occurrence of delirium and improvement of nursing quality in the PACU has rarely been reported. The aim of this study was to apply anesthesia depth monitoring, especially bispectral index (BIS), to observe patients undergoing elective surgery under general anesthesia to observe the incidence of awakening delirium and other adverse events in patients and explore the impact on the workload and quality of nursing care in the PACU. The results of this study may be used to ensure the safety of patients in the PACU and promote rapid recovery.

## Materials and methods

General information

This study was approved (no. K2020097) by the Ethics Committee of the Fourth Affiliated Hospital of School of Medicine Zhejiang University, registration of clinicaltrials.gov (ID: NCT06302517). We obtained informed consent from the patient or family. The data presented in this article were collected from September 1, 2020, to May 31st, 2022, at the Fourth Affiliated Hospital of the School of Medicine Zhejiang University.

This randomized controlled trial used the convenience sampling method to select 120 patients undergoing elective operation under general anesthesia. Inclusion criteria were: (1) age ranging from 20 to 80 years; (2) estimated operation time of two to three hours; (3) body mass index ≤30 kg/m^2^; and (4) normal preoperative heart, liver, lung, and renal function. Exclusion criteria comprised: (1) cerebrovascular disease, cognitive impairment, or prolonged use of antipsychotic drugs; (2) inability to cooperate or speak; (3) audiovisual dysfunction; (4) alcohol and drug dependence; (5) severe allergy or be prohibited from general anesthesia by endotracheal intubation, be transferred to ICU after surgery; and (6) refusal to participate in the study.

All patients were randomly divided into a BIS-guided group (group B) and a non-BIS-guided group (group C), with 60 patients in each group. An anesthesiologist monitored and recorded the BIS and vital signs of the patients during the operation, and the anesthesia nurse independently monitored, evaluated, and recorded various indicators of the patients in the PACU. 

Anesthesia and monitoring methods

All patients were routinely fasted for eight hours before anesthesia. After entering the operation room, intravenous access was established for patients without administering preanesthetic drugs. The electrocardiogram was continuously monitored using a Benevision N17 (Mindray), and blood pressure, mean arterial pressure (MAP), heart rate (HR), pulse oxygen saturation (SpO_2_), and end-expiratory partial pressure of carbon dioxide (PetCO_2_) were recorded. In group B, after removing the oil from the forehead skin with ethanol cotton balls, the BIS electrode was affixed and connected to the BIS monitoring system (BIS EEG VISTA; Covidien, Mansfield, MA, USA), recording the baseline value (T0). Anesthesia induction involved the administration of intravenous midazolam (0.02-0.2 mg/kg), sufentanil (0.25-0.5 μg/kg), and rocuronium (0.6-1.0 mg/kg), followed by intubation. Sevoflurane, intravenous remifentanil, and propofol were used to maintain anesthesia, with continuous additions of sufentanil and rocuronium throughout the operation. The BIS index of patients was monitored throughout, and the anesthesia level was adjusted according to the BIS, maintaining it within the range indicative of a general anesthesia state (40−60). In contrast, group C did not undergo BIS monitoring, and the depth of anesthesia was adjusted using traditional methods, relying on the clinical signs of the patients. The PetCO_2_ of the two groups was maintained at 30-35 mmHg during surgery. Transfusion and vasoactive drugs were administered in response to bleeding and blood pressure fluctuations. Following the surgery, the patients were transferred to the PACU for postoperative resuscitation. Patients were extubated in the operation room or PACU. The PACU team did not know the group status to ensure the blinding.

Observation indicators

MAP, HR, and SpO_2_ were closely monitored at multiple time points: at the beginning of surgery (T0), at the time of extubation (T1; when patients regained consciousness by responding to name and had spontaneous and smooth respiration, the endotracheal tube was removed) [14,15], 10 mins after extubation (T2), and at the time of leaving the PACU (T3; when patients were transferred from the PACU to the ward). The recovery period of patients was recorded, including time to extubation and PACU observation time. 

Adverse events during the PACU stay were recorded, including postoperative delirium, nausea, vomiting, respiratory complications (e.g., SPO_2_ < 90%, arterial oxygen partial pressure < 60 mmHg, or interventions requiring mask ventilation, oxygen supply, manual mandibular ventilation, and intubation), cardiovascular-related complications (e.g., blood pressure exceeding 20% of preoperative value, HR ＞120 beats/min or < 50 beats/min, emerging arrhythmia, and ischemia), severe pain, chills/hypothermia (skin temperature < 35°C), and unplanned transfer to ICU.

Delirium was assessed using the Ricker sedation-agitation scale (SAS; unable to awaken = 1, very calm = 2, calm = 3, quiet cooperation = 4, agitated = 5, very agitated = 6, and dangerously agitated = 7), and the maximum score for each patient was recorded. The evaluators were trained before the study to make them familiar with the clinical symptoms of delirium and how to use the scale. The patients were scored when they met the PACU transfer criteria.

The nursing activity score (NAS), developed by Fasoi et al. [16], consists of 23 nursing items in five aspects, including monitoring health care, supporting patients and their families, and managing nursing administration. Each item was assigned a score of 1.2−32.0 according to the percentage of time spent on the task during a nurse’s working day. The higher the score, the greater the workload. The NAS score of each patient ranged from 0 to 177, and the assessment was conducted at the beginning of resuscitation in the PACU by a trained investigator according to the nursing work items on the NAS scale.

The patients were followed up 24 hours after surgery to inquire about the comprehensive satisfaction score for anesthesia and nursing. The satisfaction score was categorized as excellent or generally poor based on criteria such as postoperative pain, influence on daily activities, and emotional stability. Excellent satisfaction indicated no or mild pain after surgery, no interference with daily activities, and emotional stability. General satisfaction indicated moderate pain that was tolerable, daily activities that were not affected or slightly reduced, and emotional stability. Poor satisfaction indicated moderate pain, significantly limited patient activities, low mood, or irritability.

Randomization and blindness

Using random numbers created by computers, 120 patients were randomly divided evenly between two groups (group B and group C). A nurse not involved in the trial used sealed, opaque, and sequentially numbered envelopes to ensure random assignment. Observers, cases, and outcome evaluators were all unaware of the experimental process.

Sample size calculation and statistical analysis

Based on a meta-analysis of randomized trials, we assumed a 40% incidence of postoperative delirium in group C and the incidence of delirium under BIS monitoring to be more than one-third lower [17-19], we assumed a 15% incidence. Set α = 0.05 on both sides, and the assurance is 80%. Calculate the sample size according to the following calculation formula: n = (2p̅q̅ (Zα+Zβ)²)/(P₁-P₂)² n = 51 cases were obtained. Considering 1:1 randomized grouping, that is, 51 cases were required in each of the intervention group and the control group. Consider 15% of missed and denied visits. Finally, at least 60 cases in each group were required, and a total of 120 cases were included.

SPSS 26.0 (IBM Corp., Armonk, New York, USA) was used to conduct the statistical analysis. Measurement data are expressed as mean ± standard deviation (x̄ ± s). Continuous variables, depending on their distributions, are presented as the mean (SD). An independent sample t-test was used for comparison between groups of data, whereas the chi-square test was used for count data, with a significance level set at P < 0.05.

## Results

**Figure 1:**
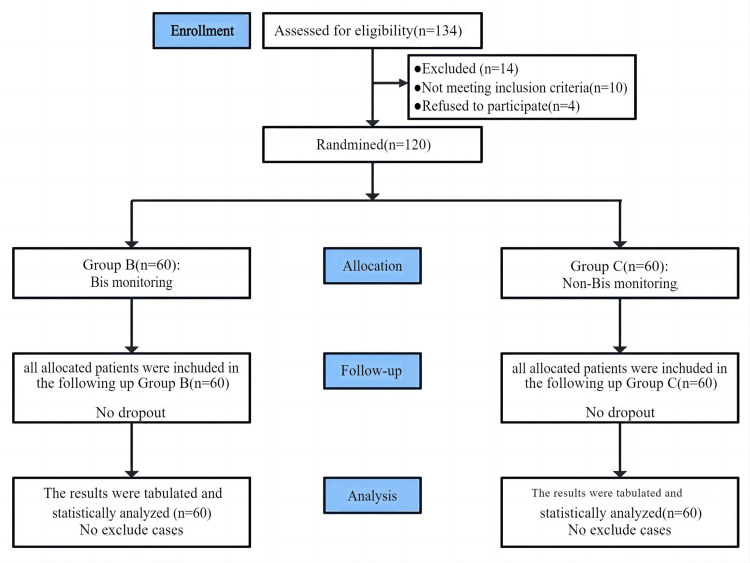
CONSORT flowchart of the enrolled patients. CONSORT: Consolidated Standards of Reporting Trials.

Ten patients did not meet the requirements, and four patients refused to participate in the experiment out of 134 that were deemed eligible. The remaining 120 cases with an allocation ratio of 1:1. (60 cases in each) were randomly divided into two groups. Statistical analysis was performed on all assigned cases (Figure 1). The retention rate of study in the intervention group and the control group was 100%. There was no harm to patients in this study. No adverse events were observed during the intervention period. 

There was no significant difference in the general condition between the groups (P ≥ 0.05), as detailed in Table 1.

**Table 1:** Comparison of the general conditions between the two groups (x̄ ± s).

Characteristic		Group B (n = 60)	Group C (n = 60)	P-value
Age (years) (mean, SD)		51.65 ± 17.13	51.46 ± 17.20	0.953
Sex (n, %)	Male	27 (45)	28 (46.7)	0.855
	Female	33 (55)	32 (53.3)	
Height (cm) (mean, SD)		164.6 ± 8.29	164.0 ± 6.99	0.686
Weight (kg) (mean, SD)		56.9 ± 11.08	54.8 ± 9.59	0.854
American Society of Anesthesiologist physical status classification system (n, %)	Ⅰ	22 (36.7)	25 (41.7)	0.567
	Ⅱ	21 (35)	20 (33.3)	
	Ⅲ	17 (28.3)	15 (25)	
Type of surgery (n, %)	Thoracic	5 (8.33)	6 (10)	1.0
	Gastric	6 (10)	7 (11.7)	
	Hepatobiliary and pancreatic	7 (11.7)	6 (10)	
	Intestinal	5 (8.33)	6 (10)	
	Head and neck	7 (11.7)	6 (10)	
	Breast	7 (11.7)	8 (13.3)	
	Urological	7 (11.7)	6 (10)	
	Orthopedic	6 (10)	6 (10)	
	Gynecological	7 (11.7)	7 (11.7)	
	Others	3 (5)	2 (3.33)	

Comparison of surgical anesthesia-related conditions between the groups showed no significant differences in operation time, anesthesia time, blood loss, fluid volume, and other surgery-related conditions (P > 0.05; Table 2).

**Table 2:** Comparison of anesthesia-related conditions between the groups (x̄ ± s).

Group	Operation time (min)	Anesthesia time (min)	Blood loss (mL)	Fluids (mL)
B	143.6 ± 18.35	170.6 ± 27.56	114.3 ± 88.13	1453.3 ± 453.8
C	144.4 ± 15.86	178.1 ± 21.75	119.7 ± 101.43	1401.7 ± 362.0
t-value	0.27	1.67	0.31	0.69
P-value	0.786	0.098	0.759	0.492

Comparison of vital signs of patients showed that the HR and MAP of the two groups were significantly higher at T1 and T2 than at T0 in the same group (P < 0.05). The HR and MAP in group B were significantly lower than those in group C at T1 and T2 (P < 0.05), but there was no significant difference in SpO_2_ between these groups (P > 0.05; Table 3).

**Table 3:** Comparison of vital signs between the groups in the PACU (x̄ ± s). *Compared with T0, P < 0.05; Δ compared with group C, P < 0.05. HR: heart rate; MAP: mean arterial pressure; SpO_2_: pulse oxygen saturation; PACU: post-anesthesia care unit.

Vital signs	Group	T0	T1	T2	T3
HR (beats/min)	B	73.3 ± 6.56	84.8 ± 7.0*Δ	79±6.66*Δ	74.1 ± 6.37
	C	74.1 ± 8.15	94.8 ± 8.79*	84.7 ± 8.64*	79.7 ± 8.52
MAP (mmHg)	B	74.1 ± 15.6	84.9 ± 7.38*Δ	79.6 ± 7.22*Δ	74.8 ± 7.51
	C	74.5 ± 9.11	89.9 ± 9.10*	83.6 ± 8.98*	77.1 ± 9.23
SpO_2_ (%)	B	96.9 ± 1.96	96.8 ± 1.17	97.2 ± 1.81	94.9 ± 2.35
	C	96.9 ± 1.76	96.3 ± 0.95	97.3 ± 0.97	95.2 ± 1.62

A comparison of the recovery period and adverse events among patients showed that the postoperative time to extubation and PACU observation time were significantly lower in group B than in group C (P < 0.05). The incidence of postoperative delirium, nausea, vomiting, breathing, and cardiovascular complications was significantly lower in group B than in group C (P < 0.05; Table 4). Neither group experienced severe pain, chills, hypothermia, nor unplanned ICU transfers.

**Table 4:** Comparison of the recovery period and complications between the groups (x̄ ± s). *P < 0.05, compared with group C; PACU: post-anesthesia care unit.

Group	Time to extubation (min)	PACU observation time (min)	Complications (cases (%))			
			Postoperative delirium	Nausea/vomiting	Respiratory-related complication	Cardiovascular complication
B	23.3 ± 8.11*	62.1 ± 9.97*	2 (3.3)*	2 (3.3)*	1 (1.7)*	1 (1.7)*
C	31.8 ± 8.33	70.6 ±10.25	5 (8.3)	5 (8.3)	4 (6.7)	2 (3.3)

Upon meeting the PACU transfer criteria during the delirium assessment of patients, it was observed that patients aged 61−80 years exhibited significantly higher SAS scores and delirium incidence than those aged 20−40 and 41−60 years (P < 0.05). The SAS score and incidence of delirium in patients aged 60−80 years were significantly lower in group B than in group C (P < 0.05) (Table 5).

**Table 5:** Comparison of delirium assessment between the groups (x̄ ± s). *P < 0.05, compared with group C; ΔP < 0.05, compared with those aged 60−80. SAS: Ricker sedation-agitation scale.

Group	Age	Cases	SAS (anxiety self-rating scale analysis system)	Incidence of delirium (%)
C	20−40 years	19	3.84 ± 0.37Δ	0 (0)Δ
	41−60 years	20	4.05 ± 0.76Δ	1 (5)Δ
	61−80 years	21	4.52 ± 1.12	4 (19)
B	20−40 years	18	3.78 ± 0.43Δ	0 (0)Δ
	41−60 years	21	3.81 ± 0.40Δ	0 (0)Δ
	61−80 years	21	4.0 ± 0.63*	2 (9.5)*

The NAS for patients in group B was significantly lower than that for patients in group C (P < 0.05). Both groups recovered anal sphincter function within 24−26 h, and no adverse reactions, such as pruritus, occurred (Table 6). The level of excellent satisfaction among patients in group B was significantly higher than that among patients in group C (P < 0.05).

**Table 6:** Comparison of nursing quality and satisfaction between the groups ((x̄ ± s), n (%)). * P < 0.05, compared with group C. NAS: nursing activity score.

Group	Cases	NAS	Excellent satisfaction (cases (%))
B	60	26.6 ± 6.53*	56 (93.3)*
C	60	31.7 ± 6.67	47 (78.3)
P-value	/	0	0.017

## Discussion

BIS monitoring can effectively control the depth of anesthesia, guide rational drug use, residual postoperative anesthesia drug concentration, improve the quality of patient recovery, and prevent the occurrence of complications during the recovery period, leading to a significant improvement in the PACU turnover rate [20,21]. After surgery under general anesthesia, the body's various systems undergo a transient state of instability. Simultaneously, the residual effects of anesthetics and muscle relaxants hinder the return of protective reflexes, often resulting in adverse events, including postoperative delirium, nausea, vomiting, hypoxemia, arrhythmia, pain, and shivering [9].

In this study, by monitoring and regulating the anesthesia depth of patients undergoing surgery and observing the occurrence of adverse events in the PACU, it was found that the BIS-guided group exhibited a significantly lower incidence of nausea, vomiting, and respiratory and cardiovascular complications than did the non-BIS-guided group. This suggests that BIS monitoring can effectively reduce the incidence of adverse events in patients undergoing general anesthesia in the PACU, improve recovery quality, and enhance patient safety throughout the perioperative period.

This study also found that the time of tracheal extraction in the BIS-guided group was significantly shorter than that in the non-BIS-guided group. This suggests that intraoperative anesthesia depth monitoring can improve PACU turnover rate and work efficiency. One plausible explanation is that the application of BIS monitoring guides the individualized dosage adjustment of anesthetic sedatives, maintaining an appropriate anesthesia depth. This can prevent insufficient and excessive intraoperative anesthetic depth and excessive stress reactions in the body, thereby reducing the occurrence of postoperative adverse events. Additionally, it can effectively reduce the number of patients exposed to excessive intraoperative anesthesia, thus shortening the respiratory function recovery time.

Delirium during recovery from general anesthesia is a form of postoperative acute brain dysfunction that can cause distress to patients. Improper treatment may lead to serious adverse events, such as accidental extubation or a fall from the bed causing trauma. Some studies have pointed out that the incidence of delirium during recovery affects the occurrence of delirium on the second day after surgery and beyond [22,23]. The diagnosis of delirium mainly depends on clinical symptoms, making the detection rate largely dependent on the sensitivity and specificity of the screening scale [24]. The results of this study showed that the incidence of delirium in patients aged 61−80 years was significantly higher than that in patients aged 20−40 years and 41−60 years within the same age group. Currently, the mechanism of postoperative delirium remains unclear, and effective targeted prevention and treatment measures are lacking. This study showed that monitoring the depth of anesthesia can reduce the incidence of delirium in elderly patients during the recovery period, indicating that it is an effective delirium prevention measure. The mechanism may be related to the monitoring of anesthesia depth under the guidance of the BIS to prevent or reduce the stress response caused by shallow anesthesia during surgery and the burst suppression of EEG caused by deep anesthesia. In contrast, BIS anesthesia depth monitoring reduces anesthesia drug overdose, potentially serving as another mechanism for reducing postoperative delirium [25].

Nursing anesthesia convalescence is an important part of ERAS practice. This study used the NAS to assess the effect of depth of anesthesia monitoring on PACU nursing workload. The NAS is a meticulously designed patient scoring system that categorizes patients and nursing activities and can accurately measure nursing workload [26]. 

This study is the first to apply the NAS to assess nursing workloads in the PACU. The study sample comprises patients undergoing elective surgery under general anesthesia, reflecting a diverse age and sex distribution, with no limitation based on the American Society of Anesthesiologists grade, offering a degree of representation in the surgical population. The results showed that the nursing workload in PACU was heavy. The average NAS score in the non-BIS-guided group was 31.7 ± 6.67, and the median score was 32.65. ICU nursing workload is traditionally measured over 24 hours, and most patients spend approximately one hour in the PACU for recovery. In this study, the average NAS score of the BIS-guided group was 26.6 ± 6.53, and the median score was 26.4, which was significantly lower than that of the non-BIS-guided group, indicating that intraoperative BIS monitoring can effectively reduce the workload of PACU nursing, as well as improve the work efficiency and quality of nursing. This improvement may be related to enhanced recovery conditions, such as reduced endotracheal tube extubation and PACU observation times, and a reduction in the occurrence of adverse events in the PACU, achieved through intraoperative BIS monitoring and anesthesia depth regulation.

These findings propose that monitoring the depth of anesthesia in patients undergoing surgery under general anesthesia can significantly reduce the workload of nurses in the PACU, make nursing work more efficient, and help provide optimal PACU nursing care for patients. In recent years, there has been an emphasis on promoting the development of ERAS and advocating PACU for patients after surgery to facilitate swift patient recovery post-surgery. This approach aims to minimize complications, improve patient comfort and prognosis, and promote collaborative efforts across disciplines to advance ERAS [27]. 

The study's limitation lies in the inadequate exploration of nursing characteristics aimed at improving nursing quality in the PACU. This pertains specifically to patients undergoing various regional anesthesia techniques across different hospital grades. Therefore, there is a need for further prospective, multi-center, and in-depth investigations.

## Conclusions

Intraoperative BIS anesthesia depth monitoring can effectively reduce the occurrence of adverse events during PACU admission, reduce the occurrence of delirium in elderly patients, and reduce the workload of nurses. This helps improve the quality of PACU nursing and can improve PACU turnover rates and work efficiency. Then, we can improve the quality of recovery, enhance the safety of patients throughout the perioperative period, and establish a foundation for promoting early and rapid recovery of patients. 
